# Npas4: A Neuronal Transcription Factor with a Key Role in Social and Cognitive Functions Relevant to Developmental Disorders

**DOI:** 10.1371/journal.pone.0046604

**Published:** 2012-09-28

**Authors:** Laurence Coutellier, Simret Beraki, Pooneh Memar Ardestani, Nay Lui Saw, Mehrdad Shamloo

**Affiliations:** Behavioral and Functional Neuroscience Laboratory, School of Medicine, Stanford University, Palo Alto, California, United States of America; Chiba University Center for Forensic Mental Health, Japan

## Abstract

Npas4 is a transcription factor, which is highly expressed in the brain and regulates the formation and maintenance of inhibitory synapses in response to excitatory synaptic activity. A deregulation of the inhibitory-excitatory balance has been associated with a variety of human developmental disorders such as schizophrenia and autism. However, not much is known about the role played by inhibitory synapses and inhibitory pathways in the development of nervous system disorders. We hypothesized that alterations in the inhibitory pathways induced by the absence of Npas4 play a major role in the expression of the symptoms observed in psychiatric disorders. To test this hypothesis we tested mice lacking the transcription factor (Npas4 knock-out mice (Npas4-KO)) in a battery of behavioral assays focusing on general activity, social behaviors, and cognitive functions. Npas4-KO mice are hyperactive in a novel environment, spend less time exploring an unfamiliar ovariectomized female, spend more time avoiding an unfamiliar male during a first encounter, show higher social dominance than their WT littermates, and display pre-pulse inhibition, working memory, long-term memory, and cognitive flexibility deficits. These behavioral deficits may replicate schizophrenia-related symptomatology such as social anxiety, hyperactivity, and cognitive and sensorimotor gating deficits. Immunohistochemistry analyses revealed that Npas4 expression is induced in the hippocampus after a social encounter and that Npas4 regulates the expression of c-Fos in the CA1 and CA3 regions of the hippocampus after a cognitive task. Our results suggest that Npas4 may play a major role in the regulation of cognitive and social functions in the brain with possible implications for developmental disorders such as schizophrenia and autism.

## Introduction

Npas4 has been implicated in the formation and maintenance of inhibitory synapses. This transcription factor belongs to the basic helix-loop-helix-PAS protein family [1], [2]. It is highly expressed in the brain, its expression is selectively induced by calcium influx in neurons, and it is transcribed in response to excitatory synaptic activity [2], [3]. Evidence strongly suggests that Npas4 plays a role in the development of inhibitory synapses by regulating the expression of activity-dependent genes, which in turn control the number of GABA-releasing synapses that form on excitatory neurons [3]. These findings suggest that Npas4 participates in the regulation of the balance between neuronal excitation and inhibition by contributing to the maintenance of the inhibitory pathways.

A balance between inhibitory and excitatory elements in brain circuits is believed to be important for maintenance of normal neuronal activity, which leads to synaptic plasticity. This balance is crucial for processing sensory information and for higher cognitive functions [4]. A deregulation of this balance has been associated with a variety of human developmental disorders [5], [6], and increasing evidence points to alterations in the inhibitory pathways (GABAergic interneurons) as a cause of schizophrenia (i.e. “glutamatergic hypothesis of schizophrenia” – [7]–[12]) and autism [13], [14]. However, the exact molecular circuits underlying the regulation of the excitatory-inhibitory balance and the contribution of this balance to the multiple symptoms defining developmental disorders is not well understood. Most investigations have focused on the molecular mechanisms underlying the functioning of excitatory synapses, while less is known about the role played by inhibitory synapses and inhibitory pathways in the development of nervous system disorders. Here, we further test the idea that elevation of the brain's susceptibility to stimulation, due to impairments in the inhibitory pathways, is responsible for the symptomatology of developmental disorders such as schizophrenia and autism.

To investigate whether alterations in the excitatory-inhibitory balance contribute to the expression of symptoms of developmental disorders, we tested mice lacking the transcription factor (Npas4 knock-out mice (Npas4-KO)) in a battery of behavioral assays. We aimed to evaluate general activity and social and cognitive functions since evidence indicates that the combination of hyperactivity, cognitive deficits and social incapacity is the primary determinant of holistic dysfunction in schizophrenic patients [15], [16]. We also studied the expression of Npas4 and c-Fos in various brain regions, including the hippocampus, after social and learning events.

## Materials and Methods

### Ethics Statement

All experiments were conducted in accordance with protocols approved by the Institutional Animal Care and Use Committee of Stanford University and were performed based on the National Institutes of Health Guide for the Care and Use of Laboratory Animals.

1. **Animals**

Behavioral testing was conducted on a total of 97 Npas4 mice. This line has been described previously [3]. Male Npas4 wild-type (WT), heterozygotes (HET), and knockout (KO) mice were obtained by HETxHET breeding at the Stanford Behavioral and Functional Neuroscience core facility (USA). Mice were housed in groups (2–6 mice per cage) and maintained on a 12-h reverse light-dark cycle with access to food and water *ad libitum*. All mice were at least 7 weeks old at the beginning of experiments, and behavioral procedures were conducted during the dark phase of the cycle. The battery of behavioral tests was performed over several weeks. KO mice were found to die at a much younger age (around 3 months of age) than WT and HET mice, which explains the absence of KO mice tested in the elevated-zero maze and the low number of KO mice tested in the novel object recognition test.

Npas4 expression in the brain after a social encounter was tested by immunohistochemistry. This experiment was conducted on male C57Bl/6j mice (n = 8) from Jackson Laboratory (Bar Harbor, ME, USA). Mice were group-housed on a 12-h reverse light-dark cycle with access to food and water *ad libitum*. They were tested at 12 weeks of age.

All experiments were conducted blind to the genotype of the mice.

1. **Test of anxiety and locomotor activity**

**a. Elevated zero-maze.** Animals (WT n = 15; HET n = 17) were tested in 8-min trials of elevated zero-maze to assess anxiety. The maze was elevated 40 cm from the ground and featured two opposite open arms and two opposite closed arms. The closed arms were characterized by 40 cm high walls. Mice were individually placed in an open arm, facing a closed arm. Their behavior was recorded via an overhead camera and the automated tracking system Ethovision. At the end of the test, the maze was cleaned with 70% ethanol. Time spent in the open arms was recorded and analyzed as an indicator of anxiety.

**b. Open-field test.** Twenty-two WT, 22 HET, and 8 KO were placed in the center of an empty novel arena (76×76 cm) with opaque walls, surrounded by privacy blinds. For 10 minutes, each animal was allowed to explore the entire arena while being tracked by Ethovision (Noldus Information Technology, Wageningen, the Netherlands). At the end of each trial, the animal was placed back in its home cage and the arena was cleaned with 10% ethanol. The total distance moved and the percent of time spent in the center of the arena (53.5×53.5 cm) were analyzed.

1. **Tests of social behavior and social memory**

**a. 6-trial social test.** The 6-trial social test was used to assess social behavior and social discrimination. It consists of presenting ovariectomized females (OEF) to each subject mouse (WT n = 10, HET n = 13, KO n = 9) over six 1-min trials separated by a 10-min inter-trial interval (ITI). During trials 1 to 4, subject mice were exposed to the same never-before-met OEF. In the 5^th^ trial, subject mice were exposed to a new never-before met OEF to test for social discrimination. During the 6^th^ trial, subject mice were exposed to the same OEF presented during trials 1 to 4. Each trial was videotaped and videos were analyzed offline by a trained experimenter. The time the subject mouse spent investigating the OEF was recorded; investigation was defined as the subject mouse making contact with the OEF.

**b. Tube dominance.** Animals were assayed in the tube dominance test as previously described [17], [18]. The Tube Dominance test assesses tendencies of social dominance and aggressive behavior in rodents without involving physical harm. Pairs of aged-matched animals from different genotypes (WT (n = 5) and KO (n = 8)) are formed. Subjects of each pair were simultaneously released facing each other into opposite ends of a clear, narrow tube. The more dominant animal forced its opponent out of the tube. When one animal had all four paws out of the tube, it was declared the loser. Each mouse was tested in 4 trials with a 10-min ITI. No subject faced the same opponent twice. The number of wins was reported as a percentage of total number of matches.

**c. Two-day social test.** This test was used to study detailed social behavior in a neutral environment. Two days prior to testing, each mouse was habituated to the testing arena (a large clean cage with bedding) 10 minutes daily. On the first day of testing, WT (n = 5), HET (n = 10), and KO (n = 8) mice were placed in the testing arena with an unfamiliar C57Bl/6 male (age-matched) for 10 minutes. No obvious body weight difference was observed; historical data from our laboratory indicate that the average body weight of our Npas4 HET and WT mice at 12 weeks old were 31.00±3.46 g and 30.60±1.94 g, respectively, while the average body weight of a C57BL/6J from Jackson Laboratory at 12 weeks is 27.82±1.58 g (http://jaxmice.jax.org/support/weight/000664.html). Twenty-four hours after the 1^st^ trial, subject animals were placed back in the testing arena with the C57Bl/6 male encountered during the 1^st^ trial. At the end of each trial the testing arena was changed for a new clean one. For each trial, the activity of the mice was recorded using an overhead camera. The behavior of the subject animals was analyzed offline using the Annotation software (Saysosoft; http://www.saysosoft.com). Detailed social behaviors were recorded: (1) active social behavior: the subject animal actively sniffs, approaches/follows, or touches the C57Bl/6 mouse; (2) avoidance of social contact: the subject animal actively avoids the C57Bl/6 mouse; (3) proximity: mice are in body contact without sniffing each other; (4) non-social behavior.

1. **Tests of cognitive abilities**

**a. Spontaneous alternation.** Spontaneous alternation, as an indicator of working memory, was assessed using a modified version of the procedure developed by Lalonde (2002) in WT (n = 21), HET (n = 22), and KO (n = 10). The apparatus consists of a gray plastic maze formed by three arms (A, B, and C) so as to form a Y shape (arm length: 40 cm; width: 8 cm; height: 15 cm). Each animal was placed in the apparatus for 8 minutes during which it was allowed to explore the entire maze. This test is based on a strong tendency in rodents to alternate arm entries, explained by their natural propensity to explore a novel environment over a recently explored one. The series of arm entries (e.g. ACBCABCBCA) was recorded using an overhead camera and videos were scored by an observer unaware of the animal's genotype. Alternation was defined as successive entry into the three arms on overlapping triplet sets (e.g. in the sequence ACBCABCBCA, five alternations were recorded). Percent alternation was calculated as the ratio of actual alternations to possible alternations (defined as the total number of arm entries minus two), multiplied by 100.

**b. Place and reversal learning.** A place and reversal learning test was used to assess for spatial memory and cognitive flexibility. Only WT (n = 15) and HET (n = 17) animals were tested; because of the inability of KO animals to swim and because of their early age of death, Npas4-KO mice were not tested in this paradigm. The test took place in a Y-maze filled with tap water. The water was made opaque using non-toxic white paint. A clear escape platform was submerged 8 mm from the water's surface and external-maze visual cues were placed around the maze.

Before the first day of testing, animals were exposed to the water Y-maze without the platform to detect a possible turning bias. Mice were released from the start arm and explored the maze for 6 30-second trials with an ITI of 15 minutes. The first arm entered (left or right) was recorded. Six entries into the same arm constituted a turning bias.

The following day, spatial place learning took place. The platform was placed at the end of the left or right arm in a counterbalanced fashion unless the mouse had a turning bias, in which case the platform was placed on the opposite side of the bias. A trial began by placing a mouse at the end of the start arm and ended when the mouse climbed onto the platform and remained there for 2 s, or after 60 s had elapsed. Mice that did not escape to the platform after 60 s were gently guided to it. Mice were then left on the platform for an extra 15 s and then returned to their home cage. Each day, animals were tested 8 times (8 trials) with an ITI of 15 minutes. A correct response was defined as reaching the platform without entry into the opposite arm. The criterion for acquiring spatial learning was seven or eight correct responses per day for three consecutive days. Once this criterion was reached, the reversal learning began after a 24-hour ITI. The same procedure as used during spatial place learning was employed during reversal learning, except the platform was placed at the end of the opposite arm. The same criterion for acquisition and the same dependent measures were used (seven or eight correct responses per day for three consecutive days).

**c. Novel object recognition.** The novel object recognition test was used to assess long-term memory. WT (n = 13), HET (n = 15), and KO (n = 3) mice were tested in a 20×40 cm arena to which they were habituated for 15-min the day prior to testing. The arena contained visual cues on two of its walls. On the first day of testing, animals were placed in the arena with 2 identical unfamiliar objects positioned 5 cm away from the walls. Animals were allowed to explore the arena and the objects during a 10-min trial (training session). Twenty-four hours later, one of the objects was changed for a new unfamiliar one while the other object was identical to the ones used during the training session. Again, animals were allowed to explore the arena and the objects during a 10-min trial (testing session). Each trial was recorded using an overhead camera. The amount of time spent sniffing and with the head within 1 cm of the objects was scored as exploration of the objects. At the end of each trial, the arena and the objects were cleaned using 70% ethanol.

1. **Test of sensorimotor gating – Pre-pulse inhibition (PPI)**

Sensorimotor gating was tested in the PPI test in WT (n = 5), HET (n = 10), and KO (n = 8) mice using a startle apparatus (Med Associates, Georgia, VT, USA). During this test, two KO mice developed severe seizures and died. Thus, only 6 KO mice were tested. Seizures in KO mice have been observed by other experimenters [3]. Each mouse was habituated to the equipment by being placed in the restraint device for 15 minutes per day for 3 days prior to the experiment. On testing day, subjects were exposed to a 70 dB white background noise for 5 minutes, and then exposed to three blocks of different startle pulses. In the first block, each animal was given five 120 dB startle pulses lasting 40 ms (no prepulses) with randomly variable inter-trial intervals of 10-20 s. In the second block, the animals encountered the same startle pulses with the addition of prepulse tones just prior to them. Each subject was given 12 exposures to each of the 6 different prepulses (0, 74, 78, 82, 86, and 90 dB), presented in random order for a total of 72 trials. The duration of the prepulse tone was 20 ms for all pre-pulses followed by a fixed interval of 100 ms (startle delay). After each prepulse tone, the animals received 40 ms of 120 dB startle pulse alone and the startle response of the animals was recorded. Inter-trial intervals for all 72 trials of Block 2 were randomly variable from 10–20 s. The maximum amplitude of the startle response in each trial was recorded for analysis. Block 1 and 2 were used for PPI measurements. For each PPI dB level (block 2), the PPI% was calculated using the following equation: [(startle response with startle pulse alone – startle response with prepulse and subsequent startle pulse)/ startle response with startle pulse alone] X 100. In the third block, each animal received 25 trials with 10–20 s randomly variable inter-trial intervals. Five different intensities of startle pulses (0, 90, 100, 110, 120 dB) lasting 40 ms were used for this block. Each animal was exposed 5 times to each intensity of startle pulses, which were given in random order. One ms after the onset of the startle stimulus, the startle response of the subject was recorded for 65 ms. This block 3 was used to assess acoustic startle. The animal holder of the apparatus was cleaned with 20% alcohol between each animal.

1. **Immunohistochemistry**

**a. Npas4 immunohistochemistry.** Eight male C57Bl/6J mice were used in this experiment. One week prior to the experiment, mice were singly-housed. One group of mice (n = 4) was placed in a social situation while the other group of mice (n = 4) was used as controls. For the social situation, each mouse was placed in a new clean cage with a never-before met male adult mouse. Each pair was left undisturbed for 10 minutes. At the end of the 10 minutes, mice were placed back in their home-cage and left undisturbed for 45 minutes. The control mice were placed individually in a new clean cage for 10 minutes. After 10 minutes, they were placed back in their home-cage and left undisturbed for 45 minutes. Animals were then anaesthetized with isoflurane and perfused transcardially with 50 ml of 0.1 M phosphate-buffered saline (pH 7.4) followed by 50 ml of fresh 4% paraformaldehyde (PFA). Brains were removed and post-fixed overnight in PFA at 4°C, placed in 30% sucrose until they sank, then frozen on dry ice and sectioned at 50 µm using a cryostat. Tissues were stored in cryoprotectant solution at −20°C until Npas4 staining was performed. For immunohistochemistry, free-floating sections were labeled with a rabbit anti-Npas4 primary antibody (dilution 1∶15,000), detected using Vectastain Elite ABC reagents (Vector Laboratories, Burlingame, CA, USA) with nickel intensified diaminobenzidine.

**b. c-Fos immunohistochemistry.** Three WT, 3 HET, and 3 KO mice were stained for c-Fos after novel object recognition, a hippocampal-dependent memory task. Animals were tested in this task as described earlier. Ninety minutes after the testing session, animals were anaesthetized with isoflurane and perfused transcardially as described earlier. Brains were treated as previously described. For immunohistochemistry, free-floating sections were labeled with a rabbit anti-c-Fos primary antibody (dilution 1∶10,000; Ab-5; Calbiochem/EMD, San Diegeo, CA, USA), detected using Vectastain Elite ABC reagents (Vector Laboratories, Burlingame, CA, USA) with nickel intensified diaminobenzidine.

**c. Image analysis.** Images were captured with a Zeiss Imager M2 microscope (Carl Zeiss Vision, Munich, Germany). The quantitative analysis of Npas4 expression in the hippocampus of C57Bl/6j mice was achieved using an optical density analysis as previously described [19]. Briefly, images were taken with a 5x objective at four different Bregma levels of the dorsal hippocampus (Bregma −1.22 mm; Bregma −1.32 mm; Bregma −1.94 mm; Bregma −2.30 mm), two different levels of the ventral hippocampus (Bregma −2.54 mm; Bregma −2.70 mm) and two different levels of the amygdala (Bregma −1.06 mm; Bregma −1.82 mm) according to the Mouse Brain Atlas of Franklin and Paxinos [20]. Images were analyzed using Image J software (http://rsbweb.nih.gov/ij/). In this program, a grey level of 0 represents black (no transmission) and 255 is white (full transmission). The measured parameters included the mean grayscale value of each drawn region of interest (CA1, CA2, CA3, and dentate gyrus [DG] in the hippocampus and medial amygdala [MeA], central amygdala [CeA], and basolateral amygdala [BLA] in the amygdala). Three measures were taken from each image capture: (1) a non tissue area that contained mounting media and coverslip but no tissue; this provided the measurement of the incident light, (2) connective tissue containing no cells to provide the measurement of background; (3) the region of interest. The optical density (OD) was derived using the formula: OD = log_10_ (incident light/transmitted light). This formula inverts the grey level scaling so that intense (dark) signal results in a high OD value. The background OD value was subtracted from each OD value measured at the region of interest to normalize the data.

The quantitative analysis of c-Fos expression in the hippocampus of Npas4 mice was achieved using the stereology method. The total number of c-Fos positive cells in the CA1, CA2, CA3, and DG of the dorsal hippocampus was quantified using the optical fractionator method [21], with assistance from the StereoInvestigator software from MBF Bioscience (Williston, VT, USA). Cells were counted in every 4 sections. Regions of interest were outlined under low magnification (5x), and c-Fos positive cells counted at high magnification (63x oil immersion). The counting criteria were determined so as to obtain a mean coefficient of error (CE, [22]) below 0.11.

1. **Statistical analysis**

Data were analyzed using the software Prism 5.01 (GraphPad Software Inc., CA, USA). All data were tested for normality using the Kolmogorov-Smirnof test and then analyzed using appropriate tests as described in the results section. Statistical significance was set at p≤0.05. All data are presented as mean ± standard error of the mean (SEM).

## Results

The behavioral results are summarized in Table 1.

**Table 1:** Summary of the results of the behavioral tests performed on Npas4 WT, HET and KO mice.

Test	Parameter	WT	HET	KO	P value	Interpretation
Elevated-Zero maze	Time in open arm (%)	21.38±3.01	22.76±1.95	N/A	ns	Npas4 KO and HET mice are not more anxious than WT. This behavior is not regulated by NPAS4
Open field	Time in center (%)	6.48±1.42	7.23±1.28	6.86±2.27	ns	
	Total distance (cm)	7095±225	7784±321	9009±681	WT vs.KOp<0.01	Npas4 KO mice are hyperactive in a novel environment.
6-trial test	Time sniffing 1^st^ trial	45.74±2.71	40.79±2.76	21.66±6.06	KO vs. WT and HET p<0.01	Npas4 KO mice spend less time sniffing an unfamiliar mouse
	Time sniffing 5^th^ trial	51.38±1.26	51.03±1.79	39.48±6.15	ns	Npas4 KO mice can discriminate between different mice
Tube Dominance	Wins (%)	12.5	N/A	71.9	p<0.001	Npas4 KO mice are more aggressive/dominant
2-Days social test	Day 1 – Time avoiding contact (%)	44.09±2.65	50.27±1.81	57.51±3.05	WT vs. KO p<0.01	Npas4 KO mice avoid contact with an unfamiliar mouse
	Day 1 – Time initiating contact (%)	36.97±4.88	31.87±3.26	27.08±2.95	ns	Npas4 KO mice initiate contact with an unfamiliar mouse
	Day 2 – Time avoiding contact (%)	27.02±7.62	45.21±4.97	37.66±7.79	ns	Npas4 KO mice do not avoid contact with a familiar mouse
	Day 2 – Time initiating contact (%)	50.28±10.95	29.98±6.49	43.75±10.36	ns	Npas4 KO mice initiate contact with a familiar mouse
Spontaneous alternation	Total arm entries	47.24±1.97	44.45±2.22	44.40±9.53	ns	Npas4 KO mice are not hyperactive in a Y-maze
	Alternation (%)	60.32±1.88	59.96±1.58	54.05±2.88	Vs. chance level: KO p>0.1	Npas4 KO mice show deficit in working memory
Place Learning	# days to criterion	4.13±0.13	4.47±0.11	N/A	ns	Npas4 HET mice don't have a deficit in spatial learning
	# errors day 1	3.87±0.62	4.71±0.62	N/A	p = 0.09 (T)	
	# errors day 2	0.80±0.38	2.47±0.74	N/A		
	# errors day 3	0.07±0.07	0.71±0.32	N/A		
Reversal Learning	# days to criterion	3.80±0.14	4.41±0.23	N/A	p = 0.04	Npas4 HET show a deficit in reversal learning
	# errors day 1	3.53±0.60	4.71±0.65	N/A	p = 0.052	
	# errors day 2	0.27±0.15	2.00±0.68	N/A		
	# errors day 3	0.07±0.07	0.65±0.41	N/A		
Novel Object Recognition	Time exploring new object (%)	59.88±2.77	36.25±9.07	55.68±3.13	p = 0.01	Npas4 KO mice show a deficit in object recognition memory
Pre-Pulse Inhibition	%PPI – 74 dB	64.11±8.12	38.36±4.10	20.44±3.22	WT vs KO p<0.01WT vs HET p<0.01	Npas4 HET and KO mice show a deficit in sensorimotor gating
	%PPI – 78 dB	70.87±7.62	48.07±5.87	30.42±2.94	WT vs KO p<0.05WT vs HET p<0.05	
	%PPI – 82 dB	71.28±6.40	54.74±4.41	37.07±3.87	WT vs KO p<0.001	
	%PPI – 86 dB	71.6±7.39	56.16±3.95	46.31±4.57	WT vs KO p<0.05	
	%PPI – 90 dB	76.11±4.08	63.14±4.03	47.88±6.81	WT vs KO p<0.01	

ns: non-significant (p>0.1); T: trend (0.05<p<0.1); N/A: data are not available

1. **Test of anxiety and locomotor activity**

**a. Elevated zero-maze.** The elevated-zero maze is used to assess the anxiety level of rodents [23]. No difference in anxiety, as measured by the percent of time spent in the open arm, was found between the WT and HET mice (WT 21.28±3.01%; HET 22.76±1.95%; t-test: p = 0.16).

**Figure 1:**
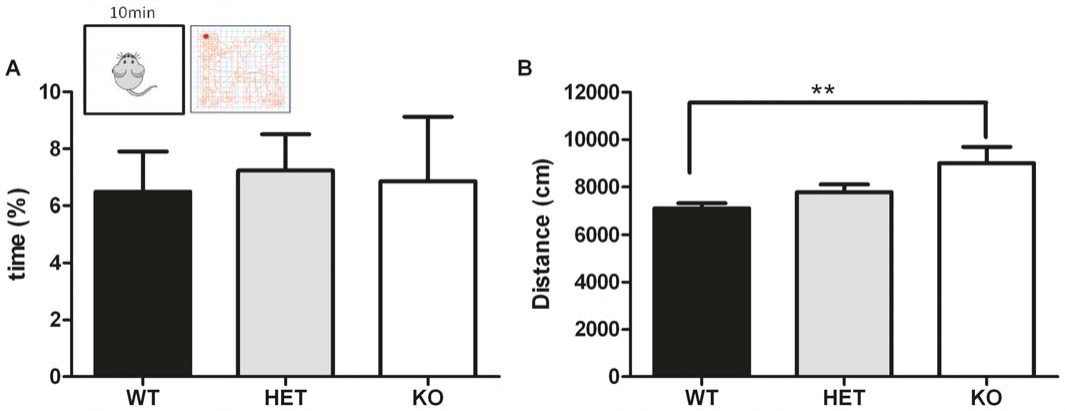
Anxiety and locomotor activity assessed in the Npas4 line. (A) Percent of time spent in the center of the open-field: no difference was observed between WT (n = 22), HET (n = 22), and KO (n = 8) mice (B) Total distance traveled in the open-field: KO mice moved a longer distance than the WT. ** p<0.01.

**b. Open-field test.** The open-field test is widely used in rodents to assess locomotor activity in a novel environment as well as anxiety [24]. The time spent in the center of the arena was not affected by the genotype of the animals (one-way ANOVA F_2,49_ = 0.08; p>0.05 – Figure 1a). The total distance traveled in the arena was significantly affected by the genotype (one-way ANOVA F_2,49_ = 5.53; p<0.01 – Figure 1b). Bonferroni post-hoc test revealed that KO mice traveled a longer distance than WT mice (p<0.01).

1. **Tests of social behavior and social memory**

**Figure 2:**
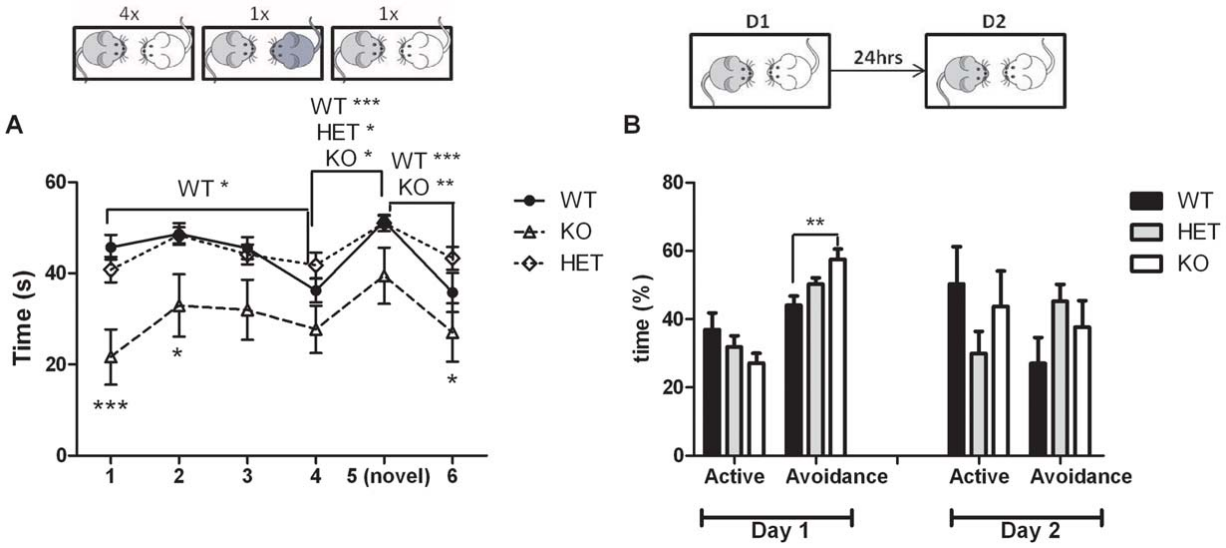
Social Behaviors assessed in the Npas4 line. (A) Time spent sniffing an unfamiliar ovariectomized female (OEF) during the 6-trial social test: KO mice (n = 9) showed less interest toward the OEF when compared to WT and HET mice (n = 10 and n = 13, respectively). However, they did not show any deficit in social discrimination as demonstrated by the increase in exploration time when they met a novel OEF (trial 5). (B) Percent of time spent in active social behavior and in avoidance behavior toward a C57Bl/6 mouse over 2 days of testing. KO mice spent more time avoiding the C57Bl/6 mouse than the WT (n = 5) during the 1^st^ day of testing. No difference was observed between HET (n = 10) and WT mice (n = 5). ***p<0.001; ** p<0.01; *p<0.05.

**a. 6-trial social test.** The 6-trial social test is commonly used to assess social investigation and social memory in mice [e.g. 25]. The time spent investigating the OEF during each of the 6 trials was analyzed using a 2-way ANOVA with repeated measures. We observed an overall genotype effect (p = 0.002 – Figure 2a). Bonferroni post-hoc tests showed that KO mice spent less time exploring the OEF than WT mice during the 1^st^ and 2^nd^ trials (p<0.001 and p<0.05, respectively) and than HET mice during the 1^st^, 2^nd^ and 6^th^ trials (p<0.01, p<0.05, and p<0.05, respectively). We also observed a significant decrease in exploration in WT mice over the 4 exposures to the same OEF (trial 1 vs. trial 4 p<0.05) while this habituation to the OEF was not seen in HET and KO mice (p>0.1). Social discrimination was assessed by presenting our subject mice with the same OEF over the first 4 trials and then introducing a never-before met OEF during the 5^th^ trial. We noted that all genotypes (WT, HET, and KO) spent more time sniffing the never-before met OEF during the 5^th^ trial when compared to the time spent sniffing the OEF during the 4^th^ trial (WT p<0.001; HET p<0.05; KO p<0.05). Finally, WT and KO mice spent less time sniffing the OEF presented during the 6^th^ trial (which was the same OEF presented during trials 1 to 4) when compared to trial 5 (WT p<0.001; KO p<0.01) while this difference was not observed in HET mice (p>0.05).

**b. Tube dominance test.** Assessment of social dominance and aggression was done based on the tube dominance test [17], [18]. Each animal was tested in 4 trials for a total of 32 WT/KO encounters. We observed that KO mice won 23 of these encounters while the WT won only 4 (5 were declared even). The distribution of wins across the two genotypes (WT 12.5%; KO 71.9% - Table 2) was found to be significantly different from a hypothetical distribution for which there would be no difference between WT and KO (i.e. 50/50 – Chi^2^ test p<0.001).

**Table 2:** Results from the tube dominance test performed in WT and Npas4 KO mice.

	WT	Npas4 KO	Even
# of win	4	23	5
% of win*	12.5	71.9	15.6

* the distribution of wins across the two genotypes is significantly different from a hypothetical distribution for which there would be no difference between WT and KO (i.e. 50/50 – Chi^2^ test p<0.001).

**c. Two-day social test.** Social investigation was assessed using a 2-trial test with a 24-hour ITI. Over the 2 days of this social test, the total time spent in social interaction was not different between the WT, HET, and KO mice (2-way ANOVA with repeated measures: F_2,20_ = 1.25; p>0.1). A time effect was observed showing a reduction in the time spent in social interaction during the 2^nd^ exposure (2-way ANOVA with repeated measures: F_1,20_ = 8.57; p<0.01). We observed a genotype effect in the percent of time spent avoiding social contact during the 1^st^ day of exposure (F_2,20_ = 6.06; p = 0.009 – Figure 2b). Bonferroni posthoc analyzes show that KO mice spent more time avoiding the unfamiliar mouse than the WT (p<0.01). This genotype difference disappeared during the 2^nd^ day of testing (p>0.1).

1. **Npas4 expression after a social encounter**

A control immunostaining was performed on a brain section from an Npas4-KO mouse. We observed a complete absence of signal. This verifies the specificity of our antibody against Npas4.

**Figure 3:**
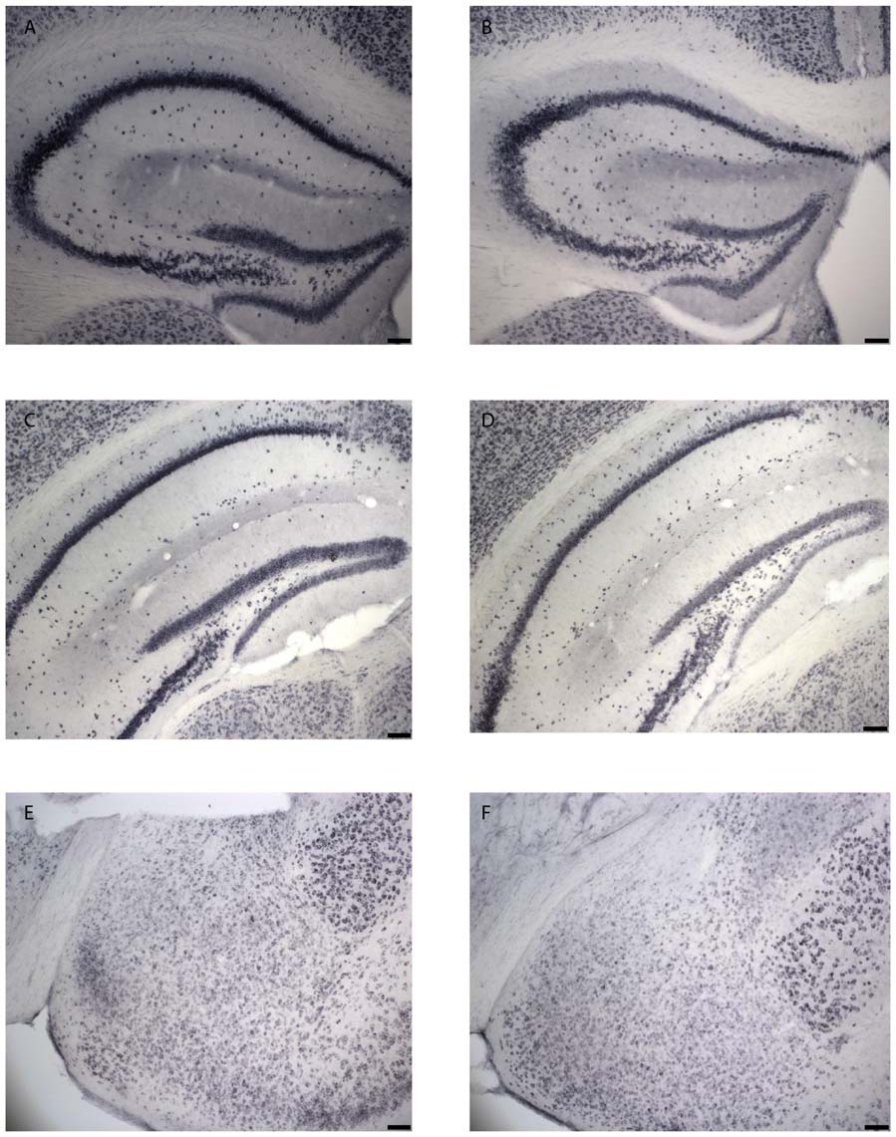
Representative pictures of Npas4 expression measured after a social interaction (A, C, E) and in control animals (B, D, F) in the dorsal hippocampus (A and B), the ventral hippocampus (C and D) and the amygdala (E and F). The scale bar on the picture represents 100 µm.

OD measurement results are presented in Table 3. OD values of the Npas4 signal in the dorsal hippocampus showed that a social encounter induced a strong Npas4 expression in the CA1 and CA3 regions of the hippocampus when compared to the expression measured in control animals (Figure 3). In the CA1 region this difference was more apparent in caudal regions of the dorsal hippocampus (Bregma −1.94 mm: t-test p = 0.03 and Bregma −2.30 mm: t-test p = 0.058). In the CA3 region, this difference was observed only at Bregma −1.34 mm (t-test p = 0.04). In the ventral hippocampus and in the amygdala no difference between the control animals and the animals exposed to the social encounter was observed (Figure 3).

**Table 3:** Npas4 expression assessed in the dorsal hippocampus, ventral hippocampus and amygdala of C57Bl/6 mice after a social interaction.

Brain region	Bregma level	Brain sub-region	Control	Social Interaction
Dorsal Hippocampus	−1.22	CA1	0.41±0.04	0.46±0.04
		CA2	0.39±0.04	0.41±0.01
		CA3	0.23±0.02	0.27±0.03
		DG	0.19±0.05	0.25±0.05
	−1.34	CA1	0.37±0.02	0.42±0.03
		CA2	0.51±0.02	0.51±0.06
		CA3	0.30±0.02	0.41±0.04*
		DG	0.26±0.03	0.29±0.05
	−1.94	CA1	0.36±0.04	0.50±0.03*
		CA2	0.42±0.07	0.49±0.02
		CA3	0.30±0.04	0.33±0.04
		DG	0.23±0.03	0.22±0.02
	−2.30	CA1	0.38±0.03	0.49±0.03^T^
		CA2	0.47±0.03	0.42±0.04
		CA3	0.37±0.02	0.38±0.03
		DG	0.24±0.02	0.26±0.02
Ventral Hippocampus	−2.54	CA1	0.34±0.32	0.37±0.02
		CA2	0.34±0.01	0.29±0.01
		CA3	0.34±0.03	0.37±0.02
		DG	0.24±0.03	0.25±0.01
	−2.70	CA1	0.36±0.01	0.36±0.01
		CA2	0.32±0.02	0.32±0.02
		CA3	0.32±0.04	0.36±0.02
		DG	0.33±0.10	0.40±0.02
Amygdala	−1.06	MeA	0.07±0.02	0.02±0.01
		CeA	0.05±0.01	0.03±0.01
		BLA	0.08±0.01	0.07±0.01
	−1.82	MeA	0.04±0.01	0.06±0.01
		CeA	0.02±0.01	0.03±0.01
		BLA	0.06±0.01	0.07±0.01

* value significantly different than the control with p<0.05; ^T^ value significantly different than the control with p = 0.058

Data are presented as the mean value of the optical density measured at various Bregma levels ± SEM.

1. **Tests of cognitive abilities**

**Figure 4:**
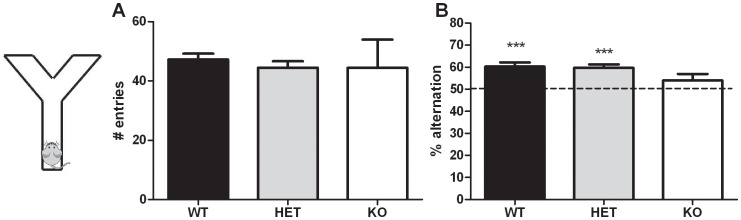
Cognitive abilities assessed in the Npas4 line. WT (n = 21), HET (n = 22), and KO (n = 10) mice were tested in the spontaneous alternation test to assess their working memory. (A) The total number of arm entries was not affected by the genotype. (B) The percent of alternation in the Y-maze was significantly above chance level (50%) in WT and HET mice but not in KO mice. *** p<0.001.

**a. Spontaneous alternation.** The Y-maze was used for assessment of spontaneous alternation for spatial working memory [26]. No genotype difference was observed in the total number of arm entries in the Y-maze (one-way ANOVA F_2,50_ = 0.21; p>0.05 – Figure 4a). We observed that both WT and HET mice have a percentage of alternation significantly above chance level (>50% – one-sample t-test: p<0.001) while this was not the case for the KO mice (p>0.1 – Figure 4b).

**Figure 5:**
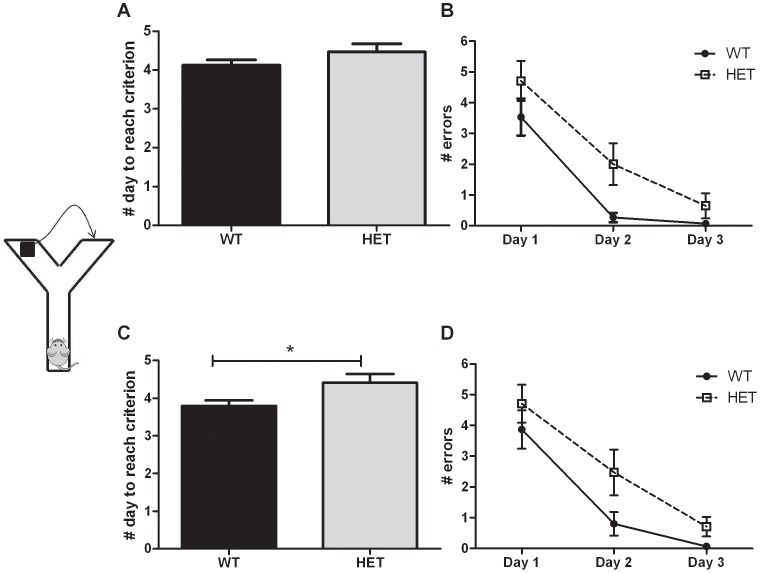
Cognitive abilities assessed in the Npas4 line. WT (n = 15) and HET (n = 17) mice were tested in a place and reversal learning task. (A) During the place learning, HET and WT mice needed the same amount of days to reach the criterion of success. However, HET mice tended to make more errors over the 3 first days of testing (p = 0.08) (B). During reversal learning, HET mice needed significantly more days to reach the criterion of success (C) and made more errors over the 3 first days of testing (p = 0.052) (D). * p<0.05.

**b. Place and reversal learning.** A water Y-maze was used to assess place and reversal learning. During place learning, the total number of days needed to reach the criterion of success was not different between the WT and HET mice (t-test p>0.1 – Figure 5a). However, the number of errors made during the first 3 days of testing tended to be higher in HET mice (2-way ANOVA with repeated measures; genotype effect p = 0.09 – Figure 5b). During reversal learning, the number of days needed to reach the criterion of success was significantly higher in HET mice compared to WT (t-test p = 0.04 – Figure 5c) and the number of errors made during the 3 first days of reversal testing was higher in HET mice than in WT (2-way ANOVA with repeated measures; genotype effect p = 0.052 – Figure 5d).

**Figure 6:**
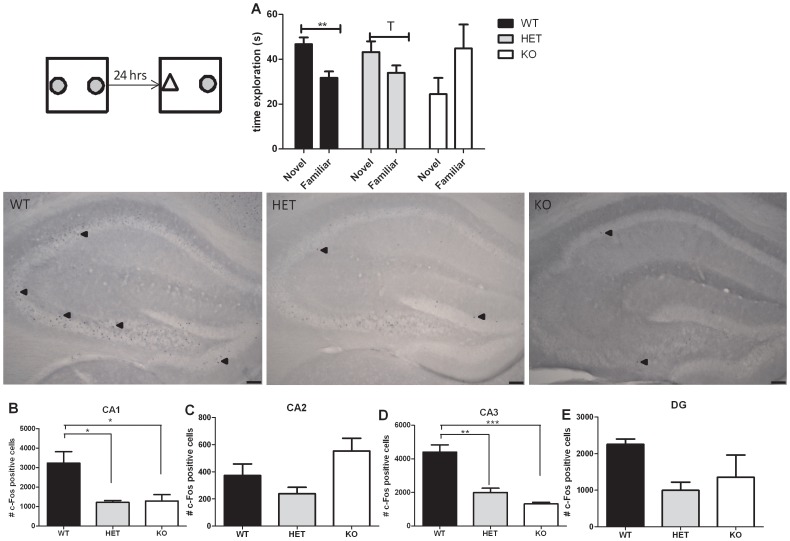
Cognitive abilities assessed in the Npas4 line. (A) During the novel object recognition test WT mice (n = 13) performed normally and sniffed a novel object longer than a familiar one; HET mice (n = 15) and KO mice (n = 3) performed poorly and did not show a preference for a novel object over a familiar one. (B-E) c-Fos expression was measured after the object recognition test in Npas4 WT (n = 3), HET (n = 3), and KO (n = 3) mice. The total number of c-Fos positive cells was counted using the stereological method in the CA1, CA2, CA3 regions and the dentate gyrus (DG) of the hippocampus. Pictures are representative pictures of c-Fos staining in the hippocampus. The black arrows represent c-Fos positive cells. The scale bar on the picture represents 100 µm. *** p<0.001; **p<0.01; *p<0.05; T p = 0.097.

**c. Novel object recognition.** Object memory in rodents is commonly assessed with the object recognition test developed by Ennaceur and Delacour [27]. In this test, WT mice performed normally, spending more time exploring the new object than the familiar one, indicating that they remembered the object presented 24 hours earlier (paired t-test p = 0.003 – Figure 6a). On the contrary, both HET and KO mice failed to remember the previously presented object; the time spent exploring the familiar object was not different than the time spent exploring the new object (paired t-test: HET p = 0.09; KO p>0.1 – Figure 6a).

1. **c-Fos expression in Npas4 WT, HET, and KO mice after a hippocampus-dependent memory task**

Results of c-Fos expression after the novel object recognition test are presented in Figure 6. We observed a high expression of c-Fos in the hippocampus of WT mice after the novel object recognition test. HET and KO mice had significantly lower c-Fos expression than WT in the CA1 region (one-way ANOVA F_2,8_ = 8.61; p = 0.02 – Bonferroni post-hoc test: WT vs. HET p<0.05; WT vs. KO p<0.05 – Figure 5b) and in the CA3 region (one-way ANOVA F_2,8_ = 29.59; p<0.001 – Bonferroni post-hoc test: WT vs. HET p<0.01; WT vs. KO p<0.001 – Figure 5d). No difference was observed between the genotypes in the CA2 region (one-way ANOVA F_2,8_ = 4.11; p = 0.08 – Figure 5c) and in the dentate gyrus (one-way ANOVA F_2,8_ = 2.88; p = 0.13 – Figure 5e).

1. **Test of sensorimotor gating – Pre-pulse inhibition (PPI)**

**Figure 7:**
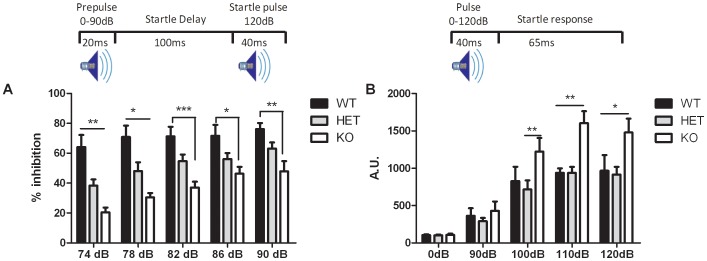
Sensori-motor gating assessed in the Npas4 line. (A) During PPI testing, HET mice (n = 10) showed less inhibition than WT with a pre-pulse of 74 and 78 dB; KO mice (n = 6) showed less inhibition than WT (n = 5) for all pre-pulse intensities. (B) During the acoustic startle response trial, KO mice showed greater startle than HET at 100, 110, and 120 dB and than WT at 110 and 120 dB. *p<0.05; **p<0.01; ***p<0.001.

Animals were tested in pre-pulse inhibition to assess their sensorimotor gating. Analysis of the acoustic startle response and PPI was done using a 2-way ANOVA with repeated measures. We first noticed that KO mice have a greater startle response than WT and HET mice (genotype effect: p = 0.01; genotype x dB: p = 0.004; dB effect: p<0.001 – Figure 7b). However, all animals from each genotype showed a strong startle response after pulses ranging from 90 to 120 dB, indicative that they hear properly (Figure 7b). In PPI, we showed a genotype effect (p = 0.001), a decibel (dB) effect (p<0.001), as well as a genotype x decibel interaction (p = 0.016 – Figure 6a). Post-hoc analysis with Bonferroni tests showed that KO mice have less inhibition than WT mice for all pre-pulse intensities (p<0.05) and HET mice showed less inhibition than WT mice for pre-pulses of 74 dB and 78 dB (p<0.05). No difference was observed between HET and KO.

## Discussion

In the present study we aimed to conduct a comprehensive behavioral analysis of mice lacking the transcription factor Npas4. Our goal was to investigate the effects of a deregulation of the inhibitory pathways caused by the absence of the transcription factor Npas4 on locomotor activity, anxiety, sensorimotor gating, social and cognitive behaviors. We observed that Npas4 null mice display several behavioral impairments, including hyperactivity in a novel environment, disruption of prepulse inhibition, social interaction deficits and cognitive impairments. These behavioral deficits may replicate the reported symptomatology of schizophrenia (social anxiety, hyperactivity, cognitive and sensorimotor gating deficits) and of autism (social and cognitive deficits). We also showed that Npas4 is highly expressed in the hippocampus in a social situation and is necessary to induce c-Fos expression in the hippocampus during a cognitive task. This suggests that the transcription factor Npas4 may play a major role in the regulation of cognitive and social functions in the brain with possible implication for neurological disorders. This supports the idea that a deregulation of the excitation-inhibition balance is responsible for a number of developmental disorders such as schizophrenia and autism [5]–[7], [11].

We first observed that mice lacking Npas4 displayed novelty-induced locomotor hyperactivity in the open-field when compared to their WT littermates. Hyperactivity is observed in many rodent models of schizophrenia [28] and is often attributed to increased dopaminergic tone [e.g. 29], [30]. However, other studies have shown that some mouse lines characterized by impaired excitatory-inhibitory balance show hyperactivity independently of dopaminergic functions [31]. With the present study we are not able to determine whether the hyperactivity observed in Npas4-KO mice is dopaminergic-dependent or a direct consequence of impairments in the inhibitory pathways. Further studies are required to answer this question.

We did not observe a significant anxious phenotype in Npas4-KO and HET mice tested in the elevated-zero maze and the open-field test. The time they spent in the open-arm of the zero-maze or in the center of the open-field did not differ from the time WT spent in these areas. These results might be surprising considering that alterations in the inhibitory pathways have been associated with increased anxiety [e.g. 7], [32], [33]. However, mice lacking Npas4 displayed another form of anxiety: social anxiety. When presented for the first time to an unfamiliar ovariectomized female, Npas4 HET and KO mice failed to habituate to this female over several exposures and Npas4 KO mice spent less time exploring it than did the WT and HET mice. Another similar paradigm supported the idea of social anxiety in mice lacking Npas4: when Npas4-KO mice faced an unfamiliar male conspecific in a neutral environment, they spent more time avoiding contact with the conspecific than the WT. This avoidance disappeared during a 2^nd^ encounter with the same conspecific. In addition to this social anxiety, we used the tube dominance test to measure another aspect of social behavior: social aggression. We noticed high aggressive/dominance behavior in the Npas4-KO mice when tested in the tube test. Pathological aggression associated with defective social interactions (i.e. social withdrawal) has been found in mouse models of mood disorders (i.e. CREB-regulated transcription coactivator 1 KO mice [34]). Also, similar social dysfunctions to the ones observed in Npas4-KO mice have already been observed in mice characterized by alterations in the inhibitory pathways such as the TgNL2.6 strain [33] or in neuregulin 1, the schizophrenia risk gene, knockout mice [35]. With the present results, we further support the idea that integrity of the excitatory-inhibitory balance in the brain is necessary for mice to display normal social behavior. Various brain regions are known to regulate social functions. Here, we observed that a social encounter between unfamiliar mice results in enhanced Npas4 expression in the dorsal hippocampus but not in the amygdala. This result suggests that Npas4 expression plays a role in social behavior in WT mice and that the lack of Npas4 might be responsible for abnormal social behavior. The hippocampus has been previously shown to be involved in social behavior. For instance, the low sociability that characterizes the BTBR mouse line has been linked with altered serotonin neurotransmission in the hippocampus [36]. Further studies have highlighted a strong relationship between serotonin and Npas4: serotonin knockout rats that display social disruptions [37], [38] show reduced Npas4 expression in the hippocampus as well as strong impairments of the GABAergic system [39]. It is thus possible that the neurotransmitter serotonin modulates social behavior by regulating Npas4 expression, which modulates GABAergic signaling. It is also possible that in absence of Npas4 (e.g. Npas4-KO mice), the modulatory effect of serotonin on social behavior is absent, leading to aberrant social interactions.

We also found that Npas4 is necessary for proper cognitive performance. Npas4-KO mice demonstrated normal short-term social memory as shown in the 6-trial test. But, they performed poorly in a task of working memory (spontaneous alternation test) and in a task of long-term memory (object recognition test). Finally, we found that Npas4-HET mice performed worse than WT mice in a test of cognitive flexibility (place and reversal learning test). Altogether, these data show that Npas4 is important for proper cognitive functioning. This is consistent with the idea that a deregulation of the excitatory/inhibitory balance has a direct impact on cognitive flexibility, working memory, and memory formation [40], [41]. Based on our data, we suggest a pathway by which the absence of Npas4 and the subsequent impairments in the inhibitory circuits affect cognitive abilities: in the absence of Npas4, we observed a strong reduction in the expression of the immediate early genes (IEG) c-Fos in the CA1 and CA3 regions of the hippocampus after the retrieval phase of an object recognition test. The role of IEG-hippocampal expression in cognitive functions is well known [42] and another study has demonstrated that Npas4 regulates the expression of c-Fos in the CA3 region after a task of long-term contextual memory [41]. With the present data we not only support this finding but we also demonstrate a similar regulatory role of Npas4 in the CA1 region in a task of long-term recognition memory. The role of the hippocampus in long-term recognition memory is well established [43], [44]. However, other brain regions such as the perirhinal cortex are also involved [45], [46]. Previous studies have shown that Npas4 is highly expressed in the cortex [2]. Thus, it would be interesting to determine whether IEG expression in this particular area is also regulated by Npas4.

Finally, in addition to hyperactivity, and social and cognitive deficits of mice lacking Npas4, we also observed an enhanced acoustic startle reflex. This high acoustic startle reflex found in Npas4-KO mice is in line with the hyperactivity previously detected. A correlation between acoustic startle response and locomotor activity has already been suggested [47]. More importantly, we showed a dose-dependent deficit in sensorimotor gating in Npas4-HET and KO mice. Npas4-KO mice displayed the strongest deficit when compared with their WT littermates and Npas4-HET mice presented an intermediate phenotype. Impaired sensorimotor gating has been proposed to be an important feature of the cognitive dysfunction observed in schizophrenia [48]. Rodent studies have revealed that disruptions in PPI are linked to stimulation of the dopamine D2 receptor, activation of the serotonergic system and/or blockade of the N-methyl-D-aspartate (NMDA) receptors [49]. In the present study, the deregulation of inhibitory synapses induced by the absence of Npas4 seems to be responsible for the deficit in PPI and for the other cognitive impairments observed in Npas4-KO mice; however, the exact mechanism is not yet known. Additional experiments will be necessary to determine the molecular pathways downstream of Npas4 by which this transcription factor regulates cognitive functions.

Altogether, our findings indicate that the expression of the Npas4 gene in the brain is directly involved in regulation of locomotor activity, sensorimotor gating, and social and cognitive behaviors in mice. The absence of Npas4 and the subsequent deregulation of the excitatory-inhibitory balance led to severe behavioral impairments that resemble those of developmental disorders characterized by a disruption of this balance. Precisely, mice lacking Npas4 display social and cognitive impairments similar to those observed in autism or schizophrenia, and hyperactivity and deficit in pre-pulse inhibition, which are endophenotypes of schizophrenia. Interestingly, Hussman [13] suggests that suppressed GABAergic inhibition is a common feature of the autistic brain. Recent advances in autism research postulates that some forms of autism are caused by an increased ratio of excitation/inhibition in sensory, mnemonic, social, and emotional systems (i.e. [14]). Similarly, several studies have reported impaired inhibitory control in the brains of schizophrenic patients [e.g. 50], [51] and deficits in episodic memory in schizophrenia have been associated with a failure of inhibitory activity in the hippocampus [52]. Finally, it is interesting to note that the location of the Npas4 gene in the human genome (chromosome 11, locus 11q13 [53]) has been associated with schizophrenia in a genome-wide linkage disequilibrium survey in a Japanese population [54]. Even if the implication of this particular locus in schizophrenia remains to be confirmed, several case studies have reported translocations between chromosome 11 and other chromosomes in schizophrenia (1, 6, 9 and 17 – see [55] for a review) supporting the idea that chromosome 11 may be highly relevant for psychosis.

To conclude, because of its unique and widespread expression in the brain, its location in the human genome and its importance in the regulation of neuronal excitatory-inhibitory signaling, the transcription factor Npas4 constitutes a plausible target for understanding the pathogenic mechanisms that underlie the complexity of diseases characterized by an imbalance between excitation and inhibition. Further studies will aim at defining the mechanism of action of Npas4 and identifying the Npas4-mediated signaling cascade in the normal brain as well as in pathological conditions such as schizophrenia and autism.
